# Biomolecular corona on nanoparticles: a survey of recent literature and its implications in targeted drug delivery

**DOI:** 10.3389/fchem.2014.00108

**Published:** 2014-11-27

**Authors:** Ryan M. Pearson, Vanessa V. Juettner, Seungpyo Hong

**Affiliations:** ^1^Department of Biopharmaceutical Sciences, University of Illinois at ChicagoChicago, IL, USA; ^2^Department of Bioengineering, University of Illinois at ChicagoChicago, IL, USA

**Keywords:** nanoparticle, biomolecular corona, targeted drug delivery, *in vivo* efficacy

## Abstract

Achieving controlled cellular responses of nanoparticles (NP) is critical for the successful development and translation of NP-based drug delivery systems. However, precise control over the physicochemical and biological properties of NPs could become convoluted, diminished, or completely lost as a result of the adsorption of biomolecules to their surfaces. Characterization of the formation of the “biomolecular” corona has thus received increased attention due to its impact on NP and protein structure as well as its negative effect on NP-based targeted drug delivery. This review presents a concise survey of the recent literature concerning the importance of the NP-biomolecule corona and how it can be utilized to improve the *in vivo* efficacy of targeted delivery systems.

## Introduction

Medical applications of nanoparticles (NPs) are wide-reaching as evidenced by their rapid development as therapeutic and diagnostic agents (Peer et al., 2007; Zhang et al., 2008; Hubbell and Langer, 2013). In particular, significant advances have been made in cancer therapy by pursuing NPs as drug delivery systems (Gu et al., 2007; Pearson et al., 2012; van der Meel et al., 2013), however, many challenges, especially with regard to achieving precise control over nano-bio interactions, still remain to be addressed (Chauhan and Jain, 2013; Pearson et al., 2014). As increasingly more complex NP formulations move toward later stages of clinical development, the need to understand and overcome those challenges is becoming imminent.

**Figure 1:**
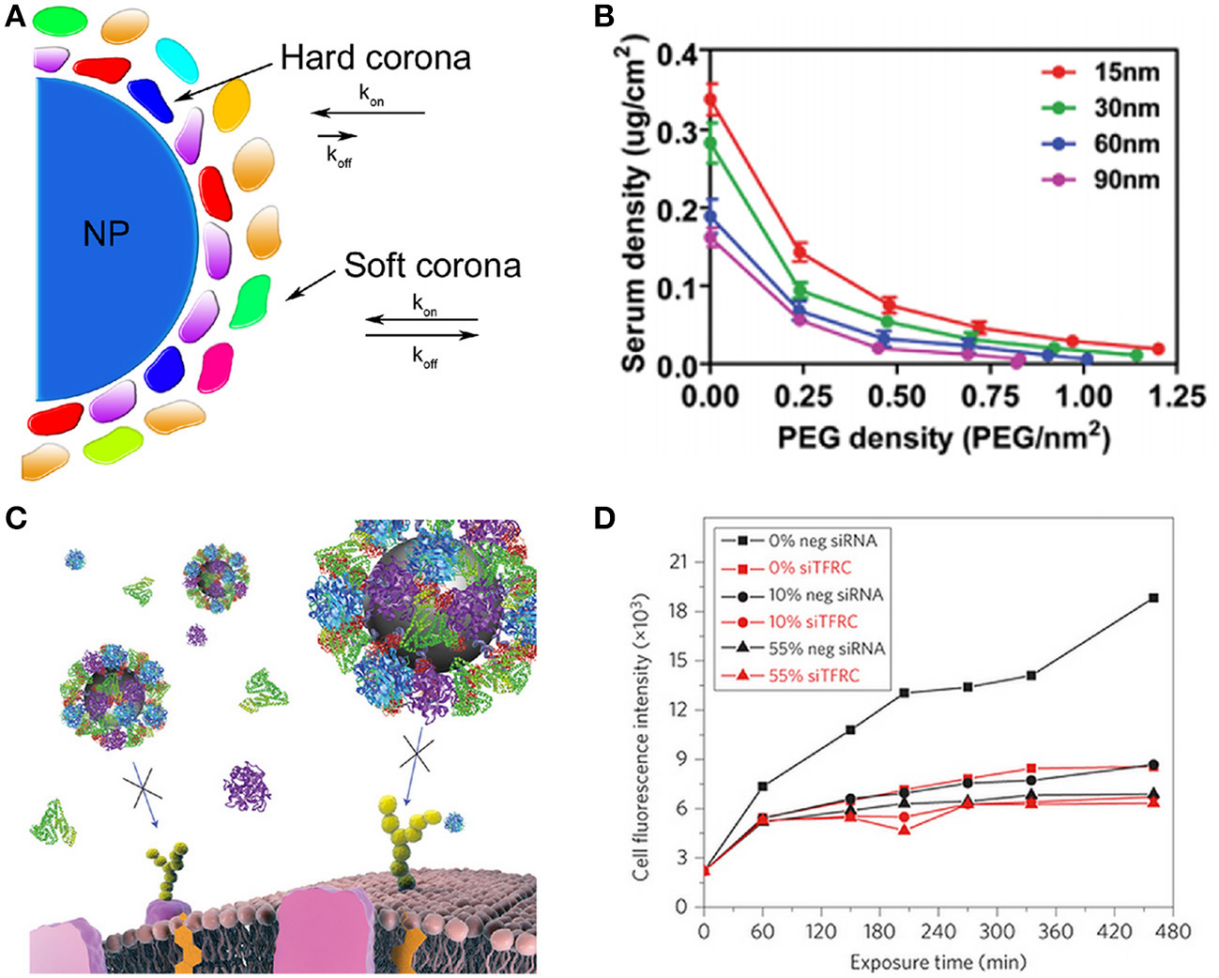
**(A)** Formation of the NP-biomolecule corona. Upon exposure to physiological fluids, NPs become coated with a variety of proteins and other biomolecules. The hard corona is comprised of lower abundance, high affinity biomolecules with almost negligible exchange rates. The soft corona is comprised of more abundant, lesser affinity biomolecules with faster exchange rates. **(B)** Size and poly(ethylene glycol) (PEG) grafting density determine PEG conformation and total serum protein adsorption to AuNPs. Reprinted with permission from Walkey et al. (2011). Copyright (2011) American Chemical Society. Negative effect of the biomolecular corona on the targeted interactions of silica NPs. **(C)** Schematic of blocked targeted cellular interactions of transferrin (Tf)-targeted NP in the presence of serum proteins. **(D)** Median A549 cell fluorescence intensity of Tf-targeted NPs in various concentrations of FBS. **(C,D)** Reprinted by permission from Macmillan Publishers Ltd: [Nature Nanotechnology] (Salvati et al., 2013), copyright 2013.

One of the most important challenges affecting NP-based drug delivery is the formation of the “biomolecule” or “protein” corona (Cedervall et al., 2007). As NPs enter physiological fluids, proteins and other biomolecules such as lipids adsorb to their surfaces with various exchange rates leading to the formation of the biomolecular corona (Figure 1A) (Nel et al., 2009; Monopoli et al., 2012; Saptarshi et al., 2013). As a consequence, the “synthetic identity” of the NP is lost and a distinct “biological identity” is acquired. This new identity governs how the NP is “seen” by cells and subsequently alters the way in which NPs interact with cells. The composition of the biomolecular corona is dynamic and is highly dependent on the initial biological environment, indicating the possibility of exposure memory (Milani et al., 2012). Opsonin adsorption such as immunoglobulin (IgG), complement, and others contribute to the deteriorated *in vivo* properties of NPs by promoting immune system recognition and rapid clearance from circulation. In contrast, dysopsonins such as albumin can coat NP surfaces and enhance their biological properties by reducing complement activation, increasing blood circulation time, and reducing toxicity (Peng et al., 2013). The binding of lipids and other lipoproteins to NP surfaces can alter the uptake and transport of NPs (Hellstrand et al., 2009). Taking these observations into consideration, the concept of the personalized biomolecular corona has arisen, suggesting that NP coronas should be characterized in a disease specific manner and not merely based on generalizations obtained from the literature (Hajipour et al., 2014).

While biomolecule adsorption alters many physicochemical properties of the NP such as size, shape, surface composition, and aggregation state, NPs may also induce conformational changes to the secondary structure of adsorbed proteins altering their biological activities (Monopoli et al., 2012). In many cases, protein adsorption to NPs can induce fibrillation, immunosensitivity, and misfolding, substantially altering properties such as biodistribution and circulation half-life, cellular uptake, intracellular localization, tumor accumulation, and toxicity (Linse et al., 2007; Aggarwal et al., 2009; Karmali and Simberg, 2011). Conversely, cases have demonstrated that biomolecule adsorption serves to protect the body from the toxicity of bare NPs, facilitating receptor-mediated interactions, and improving pharmacokinetic profiles, which demonstrates its potential advantages (Peng et al., 2013).

Fundamental forces including electrostatic interactions, hydrogen bonding, hydrophobic interactions, and charge-transfer drive the association of biomolecules to the surface of NPs (Nel et al., 2009). A recent report by Tenzer et al., found that the biomolecular corona forms almost instantaneously (in less than 30 s) and is comprised of almost 300 different proteins, although it typically consists of a similar set of proteins in various quantities (Tenzer et al., 2013). However, it has been suggested that NPs cannot accommodate as many proteins on their surfaces and a significantly lower number of proteins are present because current analyses are performed over large numbers of NPs and represent macroscopic averages of protein composition (Monopoli et al., 2012). The “hard” corona is the first layer of the corona, consisting of tightly and nearly irreversibly bound biomolecules. Atop the hard corona lie the “soft” corona layers that are composed of more leniently associated biomolecules classified by rapid exchange rates. With increasing time, less abundant, less mobile, and higher binding affinity proteins will subsequently replace the highly abundant, lower affinity proteins (Vroman effect) (Vroman et al., 1980). However, a recent study questioned the applicability of the Vroman effect to NPs and found that the composition of the hard corona was constant over time although the total amount of adsorbed proteins was changed (Tenzer et al., 2013). Properties of NPs such as size and surface hydrophobicity have also been demonstrated to affect the composition and exchange rates of proteins such as transferrin (Tf) and albumin (Ashby et al., 2014).

Although the formation of the biomolecular corona is unavoidable and plays a significant role in determining the biological behaviors of NPs, its importance has only recently received significant scientific attention. This mini review describes the importance of the NP-biomolecule corona on determining biological responses, supported by a number of recently published reports. We will succinctly cover important aspects related to biomolecular corona formation, how it is influenced by various physicochemical properties of NPs, the impact of NPs on the structure of proteins, and the impact of the biomolecular corona on the biological interactions of NPs.

## Physicochemical properties of NPs and their effect on biomolecular corona formation

The physicochemical properties of NPs determine the type of corona formed. Since the interactions between NPs and proteins occur at an interface, surface characteristics of NPs ultimately drive NP-biomolecule association. To better understand corona formation, many methods have been established (Monopoli et al., 2013; Bertoli et al., 2014). Using a bioinformatics-inspired approach, Walkey et al., developed a protein corona fingerprint model that accounts for 64 different parameters to predict the cellular interactions of NPs (Walkey et al., 2014). This model was found to be 50% more accurate than pre-existing models that only consider size, aggregation state, and surface charge. Many material properties act in concert to drive biomolecular corona formation, in this section we will focus on the effect of size, surface charge, and hydrophobicity.

It is generally accepted that a positive correlation exists for NP size and protein association. For example, a two-fold increase in protein association was measured for 110 nm silver NPs (AgNPs), compared to 20 nm AgNPs (Shannahan et al., 2013). However, an inverse correlation was also reported between the amount of mouse serum protein adsorbed and the size of 5, 15, and 80 nm AuNPs (Martin et al., 2013). It was suggested that differences in curvature enabled a larger number of hydrophobic proteins to bind to the smaller NPs in this case.

Recent reports have supported the correlation between surface charge of NPs and biomolecule association. Poly(vinyl alcohol)-coated superparamagnetic iron oxide NPs (SPIONs) with negative and neutral surface charges adsorbed more serum proteins than dextran-coated SPIONs, leading to increased circulation times (Sakulkhu et al., 2014a). Biomolecule association to PSt NPs with different sizes (50 and 100 nm) and three different surface charges [charge neutral (plain), negatively charged (carboxyl-modified), and positively charged (amine-modified)] were studied to elucidate the effect of size and surface charge of NPs on protein adsorption (Lundqvist et al., 2008). A size dependency in biomolecular corona composition was observed for both types of charged PSt NPs. For example, 100 nm negatively charged PSt NPs displayed a higher fraction of unique proteins, including Ig mu chain C region, apolipoprotein L1, and complement C1q, present in their coronas, as demonstrated by low homology in biomolecule composition compared to similar 50 nm NPs.

The connection between NP hydrophobicity and protein association has been also demonstrated to be of great importance. Isothermal titration calorimetry was used to assess the stoichiometry, affinity, and enthalpy of NP-protein interactions (Cedervall et al., 2007; Lindman et al., 2007). Titration of human serum albumin into solutions of NPs comprised of different compositions of N-isopropylacrylamine (NIPAM): N-tert-butylacrylamide (BAM), it was found that more hydrophobic NPs (50:50) bound higher numbers of albumin than more hydrophilic NPs (85:15). Larger NPs bound more albumins than smaller NP counterparts. Importantly, it was also shown that apolipoprotein A-I association was 50-fold greater for 50:50 NPs than 65:35 NPs, demonstrating favorable interactions of the proteins with the hydrophobic NPs.

Although correlations have been found with those properties, it should be noted that they could only act as predictive indicators of biomolecule association to NPs. This is important since the composition of biomolecules associated with NPs *in vitro* has been shown to be different than *in vivo* (Sakulkhu et al., 2014b). Nonetheless, the findings suggested that the surface properties of NP are responsible for driving biomolecule adsorption to the NP. Therefore, to further realize the potential of NPs as drug delivery vehicles, it is critical to coat their surface with a non-fouling layer, e.g., poly(ethylene glycol) (PEG), polyoxazoline, poly(vinyl alcohol), or polyglycerol, to minimize biomolecule association and therefore achieve more controllable cellular responses (Owens and Peppas, 2006; Romberg et al., 2008; Amoozgar and Yeo, 2012).

## Impact of PEG layers on biomolecular corona formation

Modification of the surface of NPs with a layer of PEG, or PEGylation, is known to reduce opsonization and enhance blood circulation time of NPs by providing a “stealth” effect, i.e., invisible to immune cell recognition (Owens and Peppas, 2006). Recently, a number of studies have been reported to characterize the role of the PEG conformation (i.e., brush or mushroom) and its impact on biomolecular corona formation.

The effect of PEG density on corona formation has been evaluated on numerous occasions. For example, NPs prepared from the particle replication in non-wetting templates (PRINT) method were prepared with two different PEG densities corresponding to the brush (0.083 PEG/nm^2^) and mushroom (0.028 PEG/nm^2^) regimes (Perry et al., 2012). Brush NPs displayed lower binding of bovine serum albumin (BSA) by nearly three-fold and four-fold less than non-PEGylated NPs. Significant differences between NPs with the two PEG conformations in terms of diminished macrophage uptake or increased circulation half-lives were not directly measured, but brush NPs performed better than mushroom NPs on average. At constant size, a similar result was obtained using AuNPs, where an increase in PEG grafting densities resulted in decreased serum protein adsorption (Walkey et al., 2011). In contrast, distinct differences were observed in terms of protein adsorption when size was considered. The same study found an inverse correlation between particle size and protein adsorption. The increased protein binding onto the smaller NPs was attributed to higher surface curvature and lower PEG-PEG steric interactions, which allowed a greater amount of the bare surface of the AuNP exposed (Figure 1B) (Walkey et al., 2011). When macrophage uptake was considered, two trends were observed. First, increased PEG density on similarly sized NPs resulted in decreased uptake. Second, at similar PEG densities, smaller NPs were taken up to a lesser extent than larger ones. Contrary to those results, in a study using PEGylated single-walled carbon nanotubes (SWCNT), brush SWCNTs were found to display shortened blood circulation times, faster renal clearance, and increased spleen vs. liver uptake, compared to mushroom SWCNTs (Sacchetti et al., 2013). Although these studies presented contrasting results with regard to PEG conformation, it is clear that the presence of PEG minimized biomolecular corona formation that was translated to enhanced pharmacokinetics of various NPs. However, to distinctly determine the role of PEG and PEG density in NP formulations, it is necessary to verify the biological properties of NPs in a case-by-case manner to obtain the desired response.

## Conformational changes of adsorbed proteins caused by NPs

Achieving control over the toxicity of NPs is critical to ensure their optimal therapeutic effects. When a NP enters the body, it can alter the proteins that form its protein corona, and therefore induce toxicity during therapy. Some of these changes include alterations in protein conformation, protein function, and defective transport leading to the overexpression of inflammatory factors (Baugh and Donnelly, 2003; Wolfram et al., 2014a).

Many physicochemical properties of NPs affect protein adsorption, which influences how NPs interact with cells and tissues. The proteins adsorbed on the surface of the NPs can still be recognized as the native proteins by an interacting cell, and as a result, these denatured or misfolded proteins can trigger inappropriate cellular processes (Lynch et al., 2006). In a study investigating protein stability using silica NPs, conformational changes in protein variants of carbonic anhydrase II on NP surfaces occurred in a step-wise manner, where the least stable variants exhibited the quickest misfolding kinetics (Karlsson et al., 2000). When exposed to NPs for longer periods of time, all variants eventually folded into the same unstable state.

A number of studies have been dedicated to characterizing the interaction of albumin with NPs. AuNPs, for example, modified albumin from its stable secondary conformation to its unstable tertiary conformation (Shang et al., 2007). In the case of charged-PSt NPs, it was observed that albumin maintained its native secondary structure while associated with carboxylated NPs enabling its interaction with the albumin receptor. However, amine-terminated NPs denatured albumin and subsequently led to a loss of specificity toward the albumin receptor in favor for scavenger receptors, indicating that the transition to unstable proteins alters their activity in the body (Fleischer and Payne, 2014). This illustrated that the misfolding of proteins can result in an alteration of the cell surface receptors targeted by NPs, which could decrease their targeting efficacy (Fleischer and Payne, 2014).

Mortimer et al., investigated the role of scavenger receptors in NP-protein interactions (Mortimer et al., 2014). Albumin binding to synthetic layered silicate NPs (LSNs) induced protein unfolding akin to heat denaturation of albumin. Class A scavenger receptors, which are the dominant receptors involved in the mononuclear phagocyte system (MPS), required the presence of the albumin corona to recognize the LSNs.

The conformational changes of albumin can not only lead to increased NP clearance, but also alter their cellular uptake. A study characterized the biomolecular corona of negatively charged disulfide-stabilized poly (methacrylic acid) nanoporous polymer particles (PMA_SH_ NPPs) following incubation in complete media containing 10% fetal bovine serum (FBS). Adsorption of BSA, a major component in FBS, onto the surface of the NPPs was found to result in a conformational change from its native state. Notably, denatured BSA on NPPs caused a reduction in the internalization efficiency of the NPs into human monocytic cells, compared to the bare particles, due to reduced cell membrane adhesion. However, a different conformation of BSA, triggered class A scavenger receptor-mediated phagocytosis in differentiated macrophage-like cells (dTHP-1) without a significant impact on the overall degree of cell internalization. Recognizing that both composition and orientation of the protein corona are important for the assessment of biological interactions may lead to the prevention of off target cellular interactions of NPs (Yan et al., 2013).

## Considerations of the biomolecular corona for NP-based targeted drug delivery

NP interactions with biomolecules can significantly affect the efficacies of nanomedicine. Alterations in conformations or activities of biomolecules can dramatically impair NP-based drug delivery. These alterations may result in changes in cellular uptake, drug release, and biodistribution profiles. Importantly, new methods to study those NP-cell interactions at the molecular level will yield insight into how the biomolecule corona can alter the fate of NPs (Bertoli et al., 2014).

In Table 1, we have summarized the major considerations one must take when designing and evaluating targeted NP drug delivery systems to achieve optimum efficacy.

**Table 1:** **Considerations of the biomolecular corona to design more effective NPs**.

		**References**
**PHYSICOCHEMICAL PROPERTIES OF NPs AND THEIR EFFECT ON BIOMOLECULAR CORONA FORMATION**		
Size	Larger NPs adsorb more proteins to their surfaces	Shannahan et al., 2013
Surface charge	Charged NPs adsorb more proteins to their surfaces. Alteration of particle zeta potential	Lundqvist et al., 2008
Hydrophobicity	More hydrophobic NPs adsorb more proteins to their surfaces.	Cedervall et al., 2007; Lindman et al., 2007
**IMPACT OF PEG LAYERS ON BIOMOLECULAR CORONA FORMATION**		
High density brush PEG conformation adsorbed less protein than mushroom conformations		Walkey et al., 2011; Perry et al., 2012
**CONFORMATIONAL CHANGES OF ADSORBED PROTEINS CAUSED BY NPs**		
Results in protein misfolding (changes in secondary structure)		Karlsson et al., 2000; Shang et al., 2007; Fleischer and Payne, 2014
Cryptic epitope exposure upon protein interaction with NP		Mortimer et al., 2014
Inappropriate receptor recognition after NP-protein interaction		Yan et al., 2013
**CONSIDERATIONS OF THE BIOMOLECULAR CORONA FOR NP-BASED TARGETED DRUG DELIVERY**		
*In vitro* experiments should be carried out in physiologically relevant conditions		Mirshafiee et al., 2013; Salvati et al., 2013
PEG backfilling may be used to overcome the negative effects of the protein corona on targeted drug delivery systems		Dai et al., 2014
Biomolecular corona formation can enhance pharmacokinetics of NPs		Peng et al., 2013
Biomolecular corona formation mitigates the toxicity of NPs		Ge et al., 2011; Mortensen et al., 2013

Size is an important property of NPs that affects their distribution within the body. Biomolecular corona formation can increase the original size and alter the pharmacokinetics of NPs (Lundqvist et al., 2008). In some cases, this size increase could be beneficial since NPs smaller than 5 nm are readily excreted through renal filtration (Choi et al., 2007; Sunoqrot et al., 2014). Yet, the size increase caused by biomolecule adsorption may result in a decreased therapeutic efficacy of NPs for diseases such as pancreatic cancer that require nanotherapies with particles sizes smaller than 50 nm (Cabral et al., 2011).

Considering those changes caused by the biomolecular corona, it appears essential to characterize the therapeutic and targeting efficacies of NPs under relevant conditions. Silicon dioxide (SiO_2_) NPs were functionalized with Tf to validate their ability to maintain targeted interactions in physiologically relevant cell culture conditions. In FBS-containing medium, Tf-functionalized NPs lost their ability to selectively target A549 lung cancer cells (Figures 1C,D) (Salvati et al., 2013). Mirshafiee et al., prepared 75 nm SiO_2_ NPs and studied their ability to react with synthetic, surface-bound azide groups using copper-free click chemistry (Mirshafiee et al., 2013). The results of this study confirmed that the biomolecular corona creates a barrier that screens the interaction of the ligand and its target on a separate surface.

While NP characteristics, such as size, shape, and surface charge, change due to biomolecular corona formation, drug release kinetics from the NPs can either be enhanced or disrupted. Liposomes can undergo shrinkage due to osmotic forces and may undergo a burst-release effect upon entering the blood, resulting in rapid drug release (Wolfram et al., 2014b). In contrast, protein binding on NPs has been shown to delay drug release, which prevented drug diffusion through the NP matrix (Paula et al., 2013) and reduced the burst effect (Behzadi et al., 2014).

The biomolecular corona may alter the toxicity profiles of NPs in a positive manner as well. Evidence has accumulated that the biomolecular corona may mitigate NP-induced toxicities. Decreased negative cellular impacts of carbon nanotubes were observed when they were coated with plasma proteins. Nanotubes with a higher protein density displayed less toxicity than those with a lower protein density (Ge et al., 2011). The effect of the biomolecular corona of 22 nm silica NPs with different surface charges on toxicity was also evaluated. The corona formed on each NP was confirmed to be unique, and SiO_2_-COOH NPs exhibited lower toxicity than bare SiO_2_ and SiO_2_-NH_2_ (Mortensen et al., 2013). These results indicated that NP-protein interactions can be utilized to reduce toxicities of some NPs that are otherwise known to be toxic to biological systems.

## Conclusions and future directions

The biomolecular corona has been demonstrated to have a major impact on the biological behaviors of NPs. Physicochemical properties of NPs including size, surface charge, and hydrophobicity affect the relative amounts, types, and conformations of proteins that adsorb onto the NP.

NPs functionalized with disease-specific targeting ligands are positioned to revolutionize the treatment of debilitating diseases such as cancer by achieving targeted and selective cellular interactions. However, the biomolecular corona diminishes those cellular interactions by making the ligands inaccessible at their surfaces. Therefore, development of strategies to overcome the negative impact of the protein corona on NP targeting is necessary. Recently, attaching targeting ligands to longer PEG tethers in combination with backfilling of the remaining bare surface with short PEG chains has been shown to promote the formation of targeted interactions *in vitro* (Dai et al., 2014). It is seemingly obvious that characterization and biological evaluations NPs must be performed in the presence of physiologically relevant protein levels, which will ultimately result in the enhanced *in vivo* efficacy of targeted drug delivery platforms.

### Conflict of interest statement

The authors declare that the research was conducted in the absence of any commercial or financial relationships that could be construed as a potential conflict of interest.
